# Identification of the Main Active Components and Mechanism of Wang Bi Tablet in Treating Rheumatoid Arthritis Based on Integrative Pharmacology

**DOI:** 10.3389/fphar.2021.669551

**Published:** 2021-06-04

**Authors:** Yuanyuan Jiao, Jia Xu, Hong Chen, Qiuyan Guo, Xiaofang Deng, Tong Zhang, Jingbo Zhang, Chenjing Shi, Ping Wang

**Affiliations:** ^1^ Institute of Chinese Materia Medica, China Academy of Chinese Medical Sciences, Beijing, China; ^2^ College of Traditional Chinese Medicine, Tianjin University of Traditional Chinese Medicine, Tianjin, China; ^3^ Affiliated Hospital of Guizhou Medical University, Guiyang, China; ^4^ College of Traditional Chinese Medicine, Shenyang Pharmaceutical University, Shenyang, China

**Keywords:** TCMIP, integrative pharmacology strategy, UPLC-QTOF-MS/MS, rheumatoid arthritis, Wang Bi Tablet

## Abstract

Wang Bi tablet (WBT) is used to treat rheumatoid arthritis (RA) in China. We employed integrative pharmacology, including rapid analysis of chemical composition, pharmacological experiment, and network pharmacology analysis, to elucidate the active components and mechanism underlying the effect of WBT against RA. The chemical fingerprint of WBT was revealed by UPLC-QTOF-MS/MS, and the chemical composition was identified. The anti-inflammatory effect of WBT was evaluated in TNF-α-stimulated RAW264.7 cells by ELISA and transcriptome sequencing. Network pharmacology analysis, functional enrichment analysis, and network visualization were performed. A total of 293 chemical constituents were preliminarily identified or tentatively characterized in WBT extract, and they effectively inhibited inflammatory response in TNF-α-stimulated RAW264.7 cells. Forty-eight key active constituents were identified based on high-frequency binding to hub targets and their corresponding targets number. Next, 135 corresponding hub genes, which may be the putative targets of WBT in treating RA, were selected. Functionally, the putative targets were significantly associated with the inflammatory immune response regulation module, energy metabolism regulation module, and cell function regulation module, corresponding to the traditional efficacy of WBT. In summary, this study revealed, for the first time using integrative pharmacology, that WBT may attenuate RA through the inflammation-immune regulation system.

## Introduction

Rheumatoid arthritis (RA) is a chronic autoimmune disease characterized by synovial hyperplasia, inflammatory cell infiltration, pannus formation, and destruction of articular cartilage and bone matrix, and this disease develops symmetrically and destructively, eventually leading to articular deformity and loss of function (Smolen et al., 2016; Burmester and Pope, 2017; McInnes and Schett, 2017; Aletaha and Smolen, 2018). RA has a worldwide prevalence of approximately 0.5–1%, affects women 2–3 times more often than men, and occurs in adolescents, adults, and the elderly, with a high incidence in people aged 40–60 years (Saraux et al., 2006; Myasoedova et al., 2010). Globally, the overall age-standardized prevalence and incidence rates of RA have been increasing since 1990 (Safiri et al., 2019). Between 2005–2014, the overall incidence of RA was stable compared with that in the previous decade, possibly owing to the changing prevalence of lifestyle factors, such as smoking and obesity (Myasoedova et al., 2020). As RA is a chronic, progressive disease, patients with RA have serious comorbid conditions, such as infection (Au et al., 2011), osteoporosis (Jin et al., 2018), cardiovascular disease (England et al., 2018), respiratory disease (Chatzidionisyou and Catrina, 2016), and cancer (Askling, 2007). Early diagnosis and treatment could slow down the progression of arthritic damage in 90% of RA patients, thereby preventing irreversible disability (Goekoop-Ruiterman et al., 2005). However, only a few patients can achieve long-term remission without long-term medical treatment (Matcham et al., 2014). At present, the western drugs for RA can be divided into five generations according to the development time and principles: nonsteroidal anti-inflammatory drugs, glucocorticoids, disease-modifying antirheumatic drugs, early biological agents mainly composed of TNF-α inhibitors, and new biological agents directly targeting T cells. In recent years, the development of effective biologics and small-molecule kinase inhibitors has markedly improved both the management and long-term prognosis of RA. However, these treatments are mainly used to relieve symptoms, and they cause adverse side effects including vomiting, rash, leukopenia, and liver and kidney damage. Therefore, it is urgent to find a safe and effective treatment strategy to improve both the management and long-term prognosis of RA.

Traditional Chinese medicine (TCM) is a medical and pharmaceutical system with unique theories and techniques that reflect the Chinese nations understanding of life, health, and disease. Wang Bi was first proposed by Mr. Shude Jiao in 1981 in China, mainly refers to RA and other diseases with joint deformation and bone damage, such as ankylosing spondylitis, tuberculous arthritis, and Kashin-Beck disease (Jiao and Wang, 2009). Owing to its minor side effects and reasonable treatment expenditure, TCM has become a significant strategy for treating chronic, complex, and geriatric diseases, such as RA, diabetes, emphysema, and chronic nephritis. Herbal TCMs, such as Danggui Sini decoction (Cheng et al., 2017), Guizhi Fuzi decoction (Peng et al., 2013), Huangqi Guizhi Wuwu decoction (Wang et al., 2010), and Wang Bi tablet (WBT) (Yang et al., 2009; Kang et al., 2011; Wu and Shen, 2011; Liu et al., 2012), have a long history of use in the treatment of RA. WBT is a Chinese patent medicine produced exclusively by Liaoning Haohushi Pharmaceutical (Group) Co., Ltd., and it has been recorded in the Pharmacopeia of the People’s Republic of China from the 2010 edition. WBT is composed of 17 herbal medicines, including *Rehmannia glutinosa* (Gaertn.) DC (Sheng Dihuang, SDH) 15.4g, processed *R. glutinosa* (Shu Dihuang, SD) 15.4g, *Dipsacus asper* Wall. ex DC (Xu Duan, XD) 11.5g, *Aconitum carmichaelii* Debeaux (Fu Zi, FZ) 11.5g, *Angelica pubescens* Maxim (Du Huo, DH) 7.7g, *Drynaria fortunei* (Kunze ex Mett.) J. Sm (Gu Suibu, GSB) 11.5 g, *Cinnamomum cassia* (L.). J. Presl (Gui Zhi, GZ) 7.7g, *Epimedium brevicornu* Maxim (Yin Yanghuo, YYH) 11.5g, *Saposhnikovia divaricata* (Turcz.) Schischk (Fang Feng, FF) 7.7g, *Clematis chinensis* Osbeck (Wei Lingxian, WLX) 11.5 g, *Gleditsia sinensis* Lam (Zao Jiaoci, ZJC) 7.7g, *Paeonia lactiflora* Pall (Bai Shao, BS) 9.2 g, *Cibotium barometz* (L.). J. Sm (Gou Ji, GJ) 11.5g, *Anemarrhena asphodeloides* Bunge (Zhi Mu, ZM) 11.5 g, *Lycopodium japonicum* Thunb (Shen Jincao, SJC) 7.7g, *Carthamus tinctorius* L. (Hong Hua, HH) 7.7g, and goat or sheep bones 15.4g.

Accumulating evidence from clinical practices has proved the efficacy of WBT against RA (Yang et al., 2009; Wu and Shen, 2011) and its ability to enhance the therapeutic efficacy of western medicines, such as methotrexate (Li, 2013; Zhang, 2016; Fan, 2019), in the treatment of RA. Until now, WBT research has been focused on the clinical practice. Thus, at present, basic research on WBT is still lacking in China. There is only one report on analysis of the chemical composition of WBT, in which 138 compounds were characterized by UPLC-Q-TOF-MS (Wang et al., 2019). Research on the pharmacological mechanism of WBT is limited to the NF-κB and JAK-STAT3 signaling pathways (Guan et al., 2018; Shen et al., 2019) as well as the balance between Th1 and Th2 cells (Wang et al., 2020). As a consequence, the pharmacological mechanism of WBT is largely unclear.

Integrative pharmacology, first proposed in 2014 (Xu and Yang 2014), is an interdisciplinary science that comprehensively explores the interactions between multiple constituents of TCM and the body at multiple levels, such as molecules, cells, tissues, organs, and animals (Wang et al., 2018). Integrative Pharmacology-based Network Computational Research Platform of Traditional Chinese Medicine (TCMIP v2.0, http://www.tcmip.cn/), which is composed of five databases (Xu et al., 2019) and seven functional modules (Xu et al., 2017), could assist the identification of chemical constituents and the elucidation of the molecular mechanisms of TCM (Mao et al., 2019; Feng et al., 2020; Ma et al., 2020). In the Database of TCM ingredients, we have introduced a characteristic parameter, namely “quantitative estimate of drug-likeness” (QED) to evaluate the druggability of our herbal components. QED includes ADME, water solubility, plasma protein binding rate, blood brain barrier permeability, inhibition of hepatic drug enzymes, hepatotoxicity, and intestinal absorption rate (Bickerton et al., 2012; Doak et al., 2014; Doak et al., 2016).

**FIGURE 1:**
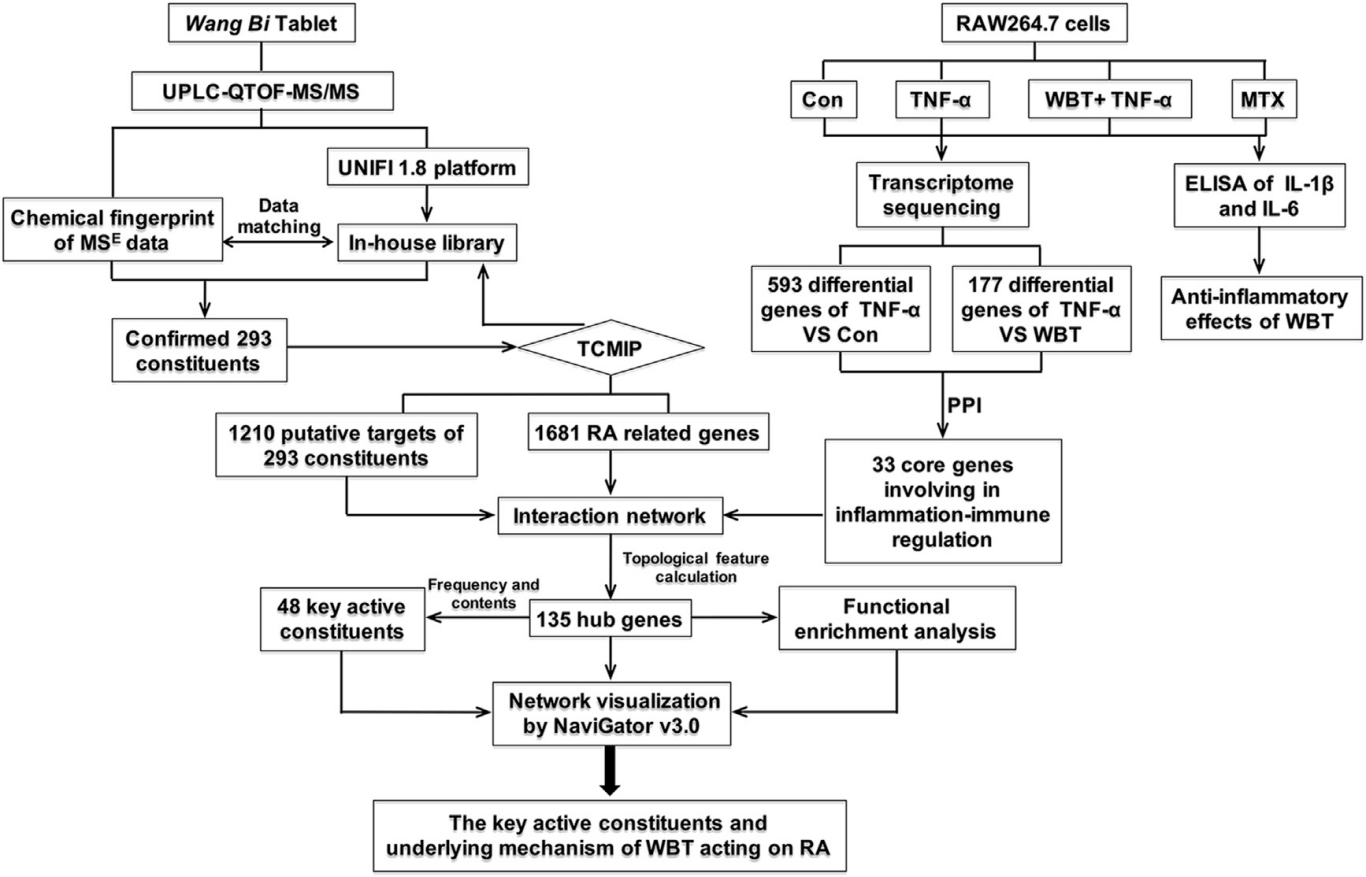
The scheme of the present study based on the “integrative pharmacology strategy” to elucidate the main active components and mechanism of WBT in treating RA.

Therefore, we aimed to systematically analyze the chemical compositions of WBT and explore its molecular mechanisms against RA through the following scheme, as shown in Figure 1: 1) analyzing the chemical components of WBT via UPLC-QTOF-MS/MS and identifying the main chemical components by using the UNIFI 1.8 software; 2) evaluating the anti-inflammatory activity of WBT in TNF-α-stimulated RAW264.7 cells via transcriptome sequencing; 3) predicting the putative targets of the identified chemical components of WBT and collecting the putative targets of RA using TCMIP; 4) constructing a network of “Anti-inflammatory Core Genes-Putative Targets-RA genes” to identify the hub genes; 5) selecting key active constituents according to the binding frequency between hub targets and WBT components, and 6) conducting a functional enrichment analysis to investigate whether the molecular mechanisms of WBT against RA is mediated via regulation of its candidate targets. The findings of this study would advance our understanding of the pharmacological mechanism of WBT against RA.

## Materials and Methods

### Chemicals and Reagents

HPLC-grade methanol and acetonitrile were purchased from Fisher Scientific Co. (Loughborough, United Kingdom). HPLC-grade formic acid was purchased from Sigma-Aldrich (St. Louis, MO, United States of America). Distilled water was purchased from Watsons Water Co., Ltd. (Shenzhen, China). SDH, SD, XD, FZ, DH, GSB, GZ, YYH, FF, WLX, ZJC, BS, GJ, ZM, SJC, HH, and goat or sheep bones were purchased from Shanghai Traditional Chinese Medicine Co., Ltd. (Shanghai, China). The herbal medicines were identified by Mrs. Xirong He, a research assistant at the China Academy of Chinese Medical Sciences (Beijing), and the voucher specimens were deposited at the Institute of Chinese Materia Medica, China Academy of Chinese Medical Sciences.

Recombinant human TNF-α (catalog #300-01A) was purchased from PeproTech, Inc (Rocky Hill, NJ, United States of America). An MTT assay kit (ab211091) as well as IL-1β (ab46052) and IL-6 (ab178013) ELISA kits were purchased from Abcam (Burlingame, CA, United States). Dulbecco's modified eagle medium (DMEM) (LOT26019006), 0.25% trypsin (LOT10519010), penicillin streptomycin solution (LOT30002341), and phosphate buffer saline (PBS) (LOT18919010) were purchased from Corning, Inc (NY, United States). Fetal bovine serum (LOT10099-141) was purchased from Gibco BRL Co. (Boise, Idaho, United States). Methotrexate was purchased from Sigma-Aldrich (St. Louis, MO, United States of America).

### Preparation of Herbal Extracts

WBT preparation was conducted in complete compliance with that recorded in the Chinese Pharmacopeia (P985-986, 2015 edition). The procedure was as follows. Briefly, SDH 15.4 g, SD 15.4 g, GSB 11.5 g, GJ 11.5 g, and goat or sheep bones 15.4 g were decocted twice in eight and six volumes of water for 1.5 h. The decoctions were filtered and then combined to obtain extract S1. The remaining 12 herbal medicines were decocted as before to obtain extract S2. Next, S2 was evaporated under reduced pressure to the weight of the original herbal medicines in a rotary evaporator and then mixed with three volumes of EtOH. The mixture was allowed to stand for 16.0 h, and then the EtOH in the supernatant was recovered under reduced pressure to obtain extract S3. S1 and S3 were combined and concentrated under reduced pressure to obtain a thick paste (S4) with a relative density of 1.27–1.30 (50°C). BS 46 g and ZM 57.5 g were ground into powder and then filtered through a 100-mesh sieve. The fine powder was mixed thoroughly with S4 before freeze drying, and the lyophilized powder was screened through a 40-mesh sieve. Six batches of herbal medicines were extracted in parallel.

The fine lyophilized powder of WBT was ultrasonically extracted using 20 volumes of 70% MeOH for 0.5 h before centrifugation at 12,000 ×*g* for 10 min in an Eppendorf 5415D centrifuge (Eppendorf Co., Hamburger, Germany). After the supernatant was filtered through a 0.22 μm filter (Pall Corporation, Beijing, China), 2 μl aliquots were transferred to the UPLC-QTOF-MS/MS system for analysis. The quality control (QC) samples of WBT were prepared by mixing the six batches of herbal medicines.

### Instrumentation and UPLC-QTOF-MS/MS Conditions

The UPLC separation was performed using a Waters Acquity UPLC HSS T3 column (100 mm × 2.1 mm, i. d. 1.8 μm) on a Waters Acquity UPLC I-Class system (Waters Corp., Milford, United States of America). The column was maintained at 40°C. The mobile phases consisted of eluent A (0.1% formic acid in deionized water, v/v) and eluent B (0.1% formic acid in acetonitrile, v/v), and the linear gradient program was as follows: 0–1 min, 1% B; 1–10 min, 1–20% B; 10–20 min, 20–40% B; 20–25 min, 40–50% B; 25–28 min, 50–100% B; 28–33 min, 100% B; 33–33.1 min, 1% B; 33.1–35 min, 1% B. The flow rate was 0.5 ml/min, and the injection volume was 2 μl.

The MS experiment was performed on a Waters Xevo G2-S Q-TOF Mass System (Manchester, United Kingdom) equipped with electrospray ionization (ESI). The data acquisition modes were MS^E^ centroid for all samples and the extra continuum mode for QC samples in both the ESI^+^ and ESI^−^ ionization modes. The operating parameters were set as follows: mass range, 50–1,500 Da; source temperature, 100°C; desolvation temperature, 400°C; desolvation gas flow, 800 L/h; sampling cone, 40 V; capillary voltage, 2.5 KV for ESI^−^, 0.5 KV for ESI^+^. At low CE scan, the auto MS collision energy was 6 eV. At high CE scan, the collision energy was 15–45 eV ramp for ESI^+^ and ESI^−^.

Leucine enkephalin was selected as the lock-mass at a concentration of 200pg/ml in acetonitrile (0.1% formic acid): H_2_O (0.1% formic acid) (50:50, v/v) for the positive ion mode ([M + H]^+^ = 556.2771) and negative ion mode ([M−H]^−^ = 554.2615) via a lock spray interface.

### Cell Culture

RAW264.7 murine macrophage-like cells were obtained from Cell Resource Center, IBMS, CAMS/PUMC (Beijing, China) and cultured in DMEM supplemented with 10% heat-inactivated fetal bovine serum and antibiotics (100 U/ml penicillin and 100 U/ml streptomycin) at 37°C in a biochemical incubator (LRH-150, Shanghai, China) with humidified atmosphere containing 95% O_2_ and 5% CO_2_.

### MTT Assay

The cytotoxicity of WBT was analyzed via MTT assay. RAW264.7 cells (1 × 10^5^ cells/well) were plated in 96-well plates (*p* = 6) and incubated for 24 h. After washing with PBS, WBT (12.8, 25.6, 128, 640, 3200, 1.6 × 10^4^, 8.0 × 10^4^, 4.0 × 10^5^, 2.0 × 10^6^, 1.0 × 10^7^ ng/ml) or PBS was added to the cells, which were then incubated for another 24 h. Cell viability was evaluated using an MTT assay kit (#ab211091; Abcam) according to the manufacturer’s protocol, and the absorbance was detected by a PerkinElmer EnVision multimode plate reader (2104, Wellesley, MA).

### Drug Treatment

RAW264.7 cells were seeded on 96-well plates (*p* = 6) at a density of 1 × 10^5^ cells per well and incubated for 24 h. Next, the medium was discarded, and the cells were washed with PBS. The cells were subsequently incubated with PBS, TNF-α (20 ng/ml), TNF-α (20 ng/ml) + WBT (0.001, 0.01, 0.1, 1.0, or 10 μg/ml), or TNF-α (20 ng/ml) + methotrexate (MTX, 0.2 μg/ml) for another 24 h. Finally, the supernatant was collected for measurement of IL-1β and IL-6 using ELISA kits according to the manufacturer’s instructions.

### RNA Extraction and Quality Control

Total RNA was isolated using a RNeasy mini kit (Qiagen, Germany) according to the manufacturer’s protocol. RNA integrity was evaluated using an Agilent 2100 Bioanalyzer (Agilent Technologies, Santa Clara, CA, United States of America). The quantity of total RNA in the samples was measured using a Qubit®3.0 Fluorometer (Thermo Fisher Scientific, United States of America) and NanoDrop One (Wilmington, DE, United States of America).

### mRNA-Seq

Paired-end libraries were synthesized using a TruSeq™ RNA Sample Preparation Kit (Illumina, United States of America) following the TruSeq™ RNA Sample Preparation Guide. Briefly, poly A-containing mRNA molecules were purified using poly T oligo-attached magnetic beads. Following purification, the mRNA was fragmented into small pieces using divalent cations at 94°C for 8min. The cleaved RNA fragments were copied into first-strand cDNA using reverse transcriptase and random primers. This was followed by second-strand cDNA synthesis using DNA Polymerase I and RNase H. These cDNA fragments then went through an end-repair process, the addition of a single ‘A’ base, and then ligation of the adapters. The products were then purified and amplified via PCR to create the final cDNA library. Purified libraries were quantified using a Qubit® 2.0 Fluorometer (Life Technologies, United States of America) and validated using an Agilent 2100 Bioanalyzer (Agilent Technologies) to confirm the insert size and calculate the mole concentration. Clusters were generated by cBot with the library diluted to 10 pM and then sequenced on an Illumina NovaSeq 6000 (Illumina, United States of America). The sequencing work was performed by Beijing Zhimei Yinuo Biotechnology Co., Ltd. (Beijing, China). The sequencing data has been uploaded to GEO website with Series record GSE165272.

### Bioinformatic Analysis

**FIGURE 2:**
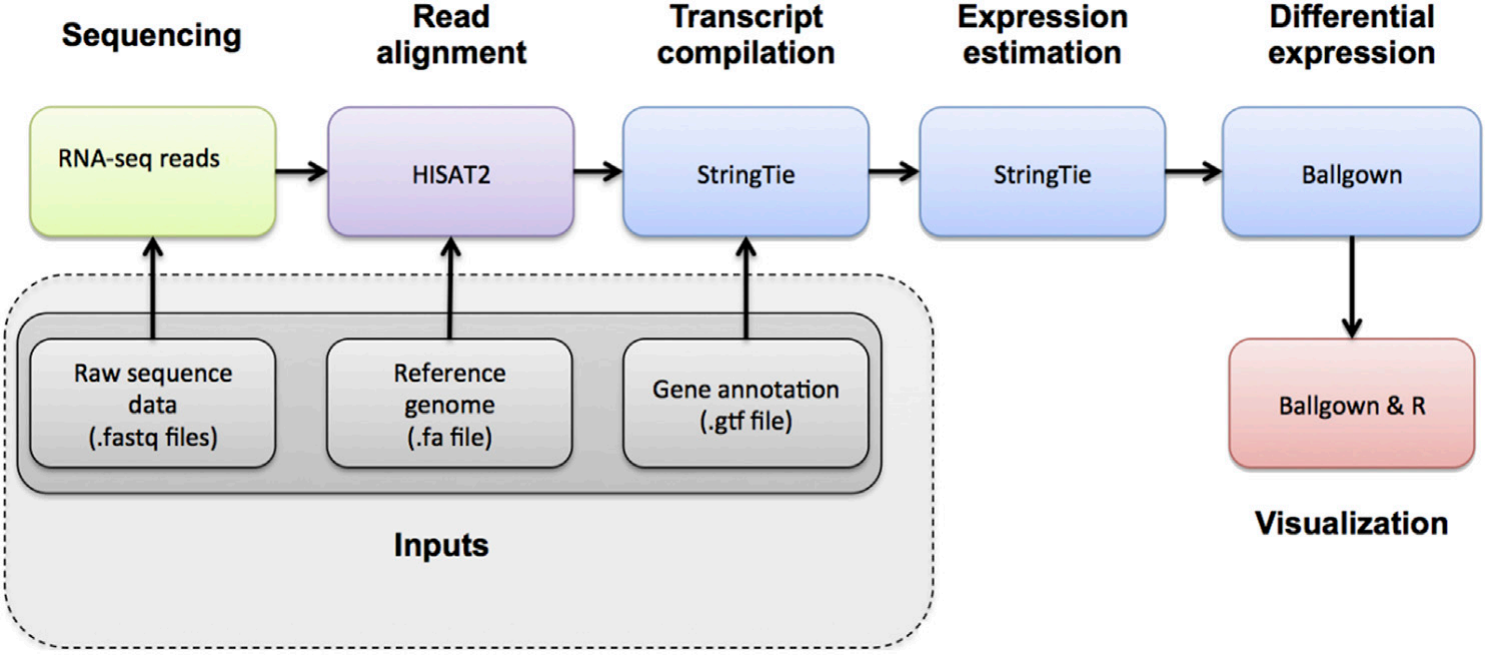
The whole procedure of bioinformatic analysis of transcriptomics.

Bioinformatic analysis was also undertaken by Zhimei Yinuo Biotechnology Co., Ltd. The whole procedure is shown in Figure 2.

### Prediction the Putative Targets of the Chemical Constituents of WBT

The TCM target prediction and function analysis module (TTFM) of TCMIP was employed to predict the putative targets of the compounds preliminarily identified in WBT. The prediction accuracy was set at 0.80 (moderate to high similarity) to select constitute-putative target pairs, and these genes were saved in the customer center. Simultaneously, the QED value was set at 0.49, and constituents with QED <0.49 will be filtered out.

### Collection of RA-Related Genes From TCMIP

The disease name “rheumatoid arthritis” and symptom nouns, such as “joint swelling”, “morning stiffness”, and “arthralgia” were selected to search in TCMIP in order to obtain RA-related genes among those saved in the personal user center.

### Network Visualization and Functional Enrichment Analysis

NaviGator v3.0 was employed to establish a network, and the Database for Annotation, Visualization and Integrated Discovery (DAVID) v6.8 (https://david.ncifcrf.gov) was used to elucidate the biological functions of the core efficacy gene set.

### Data Analysis

The UPLC-QTOF-MS/MS system was controlled by the Masslynx 4.1 software (Waters Corp.). The MS^E^ continuum data were processed by UNIFI 1.8 (Waters Corp.). The processes included data acquisition, data mining, library searching, and report generation. The chemical information of the 16 herbal medicines (except for goat or sheep bones) was collected in a list consisting of molecular name/formulae/weights and chemical structures (mol. format) from the literature and the Encyclopedia of Traditional Chinese Medicine (http://www.nrc.ac.cn:9090/ETCM/) (Xu et al., 2019). The list, as a customized library that could facilitate chemical identification, is shown in Supplementary Excel S1 [M+H]^+^, [M+K]^+^, [M+Na]^+^, [2M+H]^+^, and [M-e]^+^ were selected as additive ions in the positive ion mode. [M+COOH]^-^, [M-H]^−^, and [2M-H]^−^ were selected in the negative ion mode. The allowable maximum error range was 5 mDa/10 ppm, and the matched constituents were given the predicted fragments from the structure. The functional module of the mass fragment could facilitate the chemical identification of unmatched constituents based on the isotopic abundance, elemental composition, and i-FIT score.

## Results

### Characterization and Identification of Chemical Constituents in WBT

**FIGURE 3:**
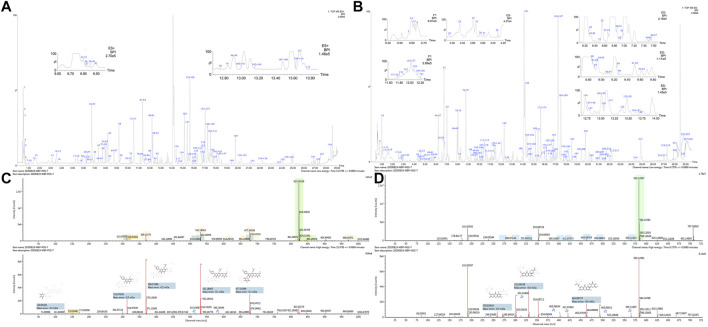
BPI chromatograms of WBT extracts **(A)** ESI^+^; **(B)** ESI^−^), and spectrum information of baohuoside VI **(C)** and akebiasaponin D **(D)** automatically provided by UNIFI™.

A total of 293 compounds were identified or tentatively characterized in both the positive (165) and negative 229) ion modes, including flavonoids, alkaloids, glycosides, coumarins, saponins, phenolic acid, and iridoids. The base peak intensity (BPI) chromatograms of WBT in two ion modes are depicted in Figures 3A,B. Among the 293 constituents characterized, 45 were identified in SDH or SD, 28 in XD, 23 in FZ, 23 in DH, 15 in GSB, 14 in GZ, 36 in YYH, 22 in FF, 7 in WLX, 8 in ZJC, 28 in BS, 8 in GJ, 26 in ZM, 16 in SJC, and 31 in HH. Detailed information of the 293 compounds is listed in Supplementary Excels S2, S3, including RT, M/Z, error, response, adducts, formula, name, fragments, category, and origin.

The BPI chromatograms of the water extract of WBT corresponding to the positive and negative ion modes are shown in Figures 3A,B. The mass spectra of two compounds are shown below. The ion at RT = 15.37 and [M + H]^+^ = 823.3022 was primarily identified as baohuoside VI (C_39_H_50_O_19_) in ESI^+^ after matching with data from the customized database (Supplementary Excel S4) of UNIFI. The main fragments were m/z 677.2438 [M + H-rha]^+^, 531.1860 [M + H-2rha]^+^, 369.1328 [M + H-Glc-2rha]^+^, and 313.0703 [M + H-Glc-2rha-C_4_H_7_]^+^, which were consistent with those in literature (Yu et al., 2016). In the same way, the ion at RT = 17.27 and [M-H]^−^ = 927.4931 was primarily identified as akebiasaponin D (C_47_H_76_O_18_) in ESI^−^. The main fragments were m/z 603.3904 [M-H-2Glc]^−^, 323.0984 [M-H-Glc-C_4_H_8_O_4_]^−^, and 179.0563 [M-H-Glc-C_3_H_6_O_3_]^−^, which were consistent with those in literature (Yang et al., 2016). The mass spectra and structures of the two compounds are displayed in Figures 3C,D.

### WBT Reduces the Release of IL-1β and IL-6 in TNF-α Stimulated RAW264.7 Cells

**FIGURE 4:**
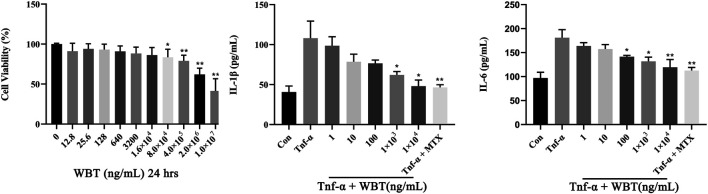
WBT prevents inflammatory response induced by TNF-α in RAW264.7 cells in a dose-dependent manner. Cell viability was determined by MTT assay **(A)**. The levels of IL-1β **(B)** and IL-6 **(C)** in cell culture supernatant were detected by ELISA kits (Mean ± SD, *p* < 0.05*, *p* < 0.01**).

The MTT assay showed that WBT exerted no significant cytotoxicity to RAW264.7 cells even at a concentration as high as 1.6 × 10^4^ ng/ml (Figure 4A). Therefore, WBT concentrations ranging from 0.001 to 10 μg/ml were selected for the examination of the anti-inflammatory activity of WBT in TNF-α-stimulated RAW264.7 cells. In RAW264.7 cells stimulated with TNF-α (20 ng/ml) for 24 h, IL-1β and IL-6 levels in the culture medium were significantly increased (*p* < 0.01) compared with that in the control group. MTX, as a positive drug, exhibited an excellent effect against inflammatory responses, suggesting a good pharmacodynamic evaluation system. In RAW264.7 cells co-cultured with TNF-α and WBT at 0.1, 1, and 10 μg/ml, IL-1β and IL-6 levels were significantly reduced (*p* < 0.01) in a dose-dependent manner. WBT at a low dose (0.001, 0.01 μg/ml) did not exhibit any anti-inflammatory effects (Figures 4B,C).

### RNA-Seq Transcriptional Comparison Between Different Groups and Functional Enrichment Analysis of Core Genes

**FIGURE 5:**
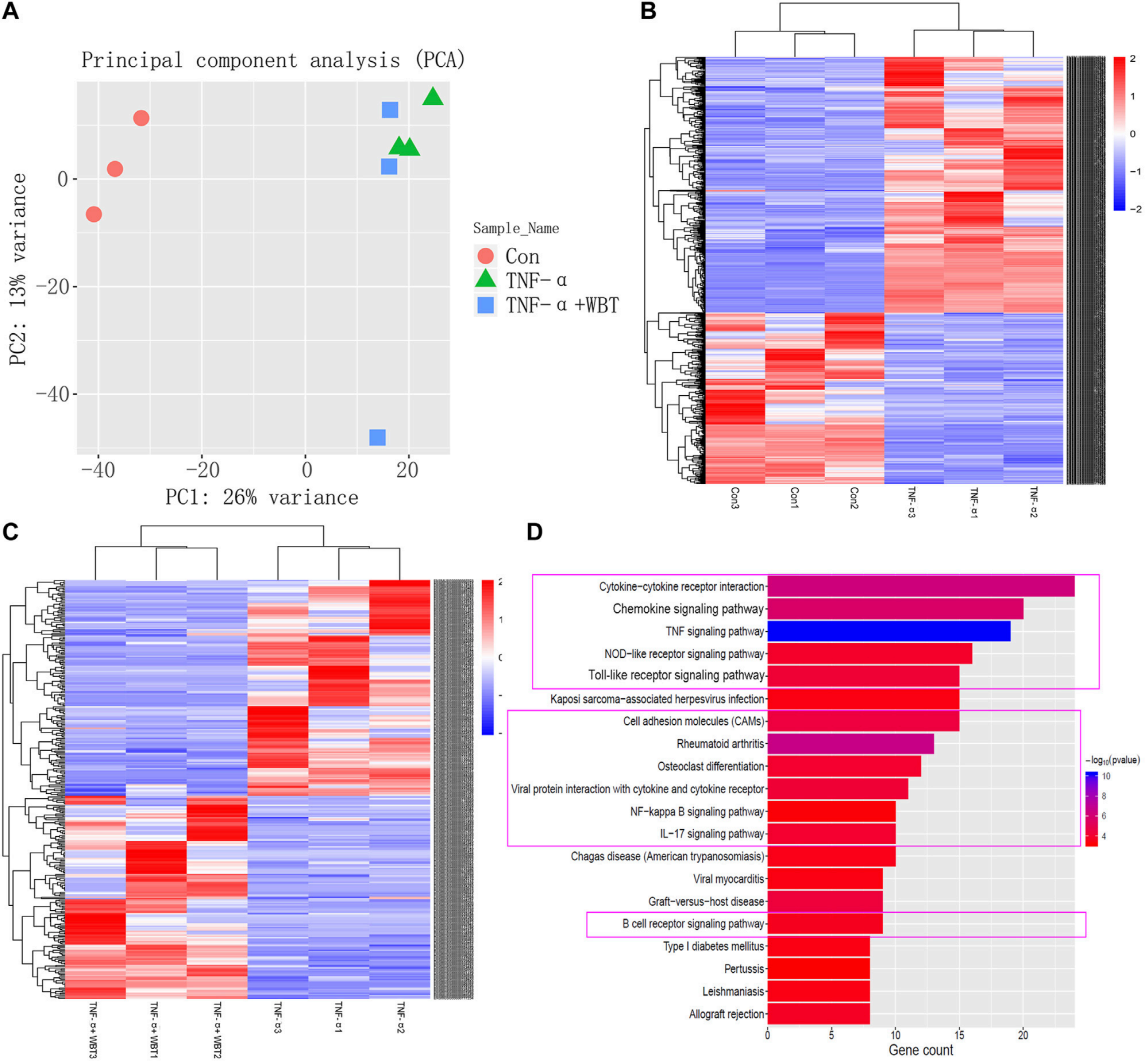
RNA-seq transcriptional comparison between different groups, and functional enrichment analysis based on network pharmacology. Principal component analysis of transcriptome sequencing results **(A)**. Differential gene expression profiles of TNF-α vs. Con **(B)** and TNF-α+WBT vs. TNF-α **(C)** were visualized in heat maps (fold change ≥2, *p* < 0.05). Pathway enrichment analysis of the hub genes responsible for the anti-inflammatory effect of WBT. The purple box indicated the pathways closely related to the occurrence and development of RA **(D)**.

To elucidate the mechanism by which WBT altered the transcriptomic profile of RAW264.7 cells simulated by TNF-α, RNA-seq experiments were performed. The control, TNF-α, and TNF-α + WBT (1.0 μg/ml) groups were selected as the biological samples according to the level of inflammatory factors. To ensure the accuracy of the analysis, we set three biological replicates for RNA-seq, a fold change of more than 2, and a *p* value of less than 0.05. Principal component analysis showed good separation between different groups and good consistency in the same group (Figure 5A). After treatment, 593 and 177 genes were differentially regulated between TNF-α vs. Con and between TNF-α+WBT vs. TNF-α, respectively (Supplementary Excel S5, S6). Between TNF-α vs. Con, 376 genes were upregulated, and 217 genes were downregulated; these genes were regarded as “Inflammatory Immune Dysregulation Genes.” Between TNF-α+WBT vs. TNF-α, 92 genes were upregulated, and 85 genes were downregulated; these genes were regarded as “Anti-inflammatory Effect Genes.” Heat maps were generated to visualize the gene expression patterns in the three groups (Figures 5B,C). Interestingly, 45 genes that were differentially expressed between TNF-α vs. Con were significantly counter-regulated by WBT; thus, these genes may be the potential gene set responsible for the efficacy of WBT in reducing inflammatory responses. The 593 and 177 different genes were imported into the STRING database for construction of a PPI network including 264 nodes and 599 edges (Supplementary Excel S7). Thirty-three core nodes of the network were obtained by calculating the topological feature values, including degree, betweenness, and closeness. Generally, the node with a degree value of two-fold the median as well as betweenness and closeness values of one-fold the median is selected as the hub node. The 33 hub nodes included ABL1, ACTG2, ALDH1A7, B2M, C3, CCL2, CCL20, CCL5, CCL9, CES2C, CSF1, CXCL1, CXCL10, CXCR3, EPHA3, EPHA5, H2-K1, HVCN1, IRF7, JUN, MFI2, MMP3, MMP9, NMUR1, OLFM4, PLXNB3, RAB27A, RHBDF2, SEMA3G, THY1, TLR2, TRF, and VCAM1. Based on functional enrichment analysis, these 33 genes were mainly involved in the inflammation-immune regulation module, and the purple box indicates that the pathway information was closely related to the occurrence and development of RA (Figure 5D), such as cytokine-cytokine receptor interaction, chemokine signaling pathway, TNF signaling pathway, NOD-like receptor signaling pathway, and Toll-like receptor signaling pathway.

### Underlying Mechanism and Key Active Components of WBT in Treating RA

By using the TTFM of TCMIP, we predicted a total of 1,210 putative targets based on the chemical structures of the 293 identified compounds (Supplementary Excel S8). Moreover, a total of 1681 RA-related genes were collected from the Disease-related Gene Database of TCMIP, as shown in Supplementary Excel S9. To illustrate the underlying mechanisms of WBT against RA, an interaction network of “Anti-inflammatory Hub Genes-Putative Targets- Disease Genes” was constructed using the TCM Association Network Mining Module of TCMIP. The interaction network included 2,251 nodes and 53,254 edges, with a network density of 0.011. A total of 566 core nodes of the network were obtained by calculating the topological feature values. In addition, a PPI network of the 566 core nodes was constructed, which included 566 nodes and 23,558 edges and had a network density of 0.074, which was significantly higher than that of the initial network (density 0.011) and in accordance with network centrality of hub nodes. Finally, 135 hub nodes were obtained by calculating the topological feature values, of which 81 were putative targets of WBT corresponding to 225 chemical constituents.

**FIGURE 6:**
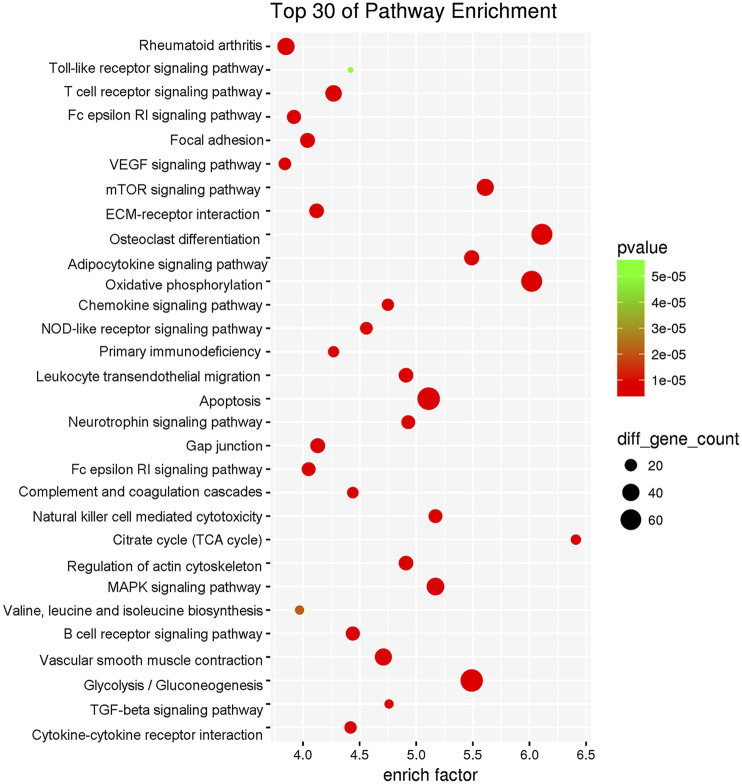
Top 30 enriched pathways for the 135 hub genes in the effect of WBT against RA.

To explore the biological function of these 135 hub genes, DAVID v6.8 was employed for KEGG analysis. As shown in Figure 6, the pathway information could be divided into five function modules: 1) the synovial inflammation-immune imbalance regulation module. It was the most significant functional module that included NOD-like receptor signaling pathway, B cell receptor signaling pathway, T cell receptor signaling pathway, Fc epsilon RI signaling pathway, Toll-like receptor signaling pathway, primary immunodeficiency, leukocyte transendothelial migration, chemokine signaling pathway, complement and coagulation cascades, natural killer cell-mediated cytotoxicity, regulation of actin cytoskeleton, Fc gamma R-mediated phagocytosis, and cytokine-cytokine receptor interaction; 2) energy metabolism regulation module, including citrate cycle (TCA cycle), oxidative phosphorylation, adipocytokine signaling pathway, neurotrophin signaling pathway, insulin signaling pathway, valine, leucine and isoleucine biosynthesis, glycolysis/gluconeogenesis, and pyrimidine metabolism; 3) cell function regulation module, including focal adhesion, mTOR signaling pathway, ECM-receptor interaction, apoptosis, TGF-beta signaling pathway, and MAPK signaling pathway; 4) synovial pannus formation module, including VEGF signaling pathway and vascular smooth muscle contraction; 5) bone destruction regulation module, including osteoclast differentiation.

**FIGURE 7:**
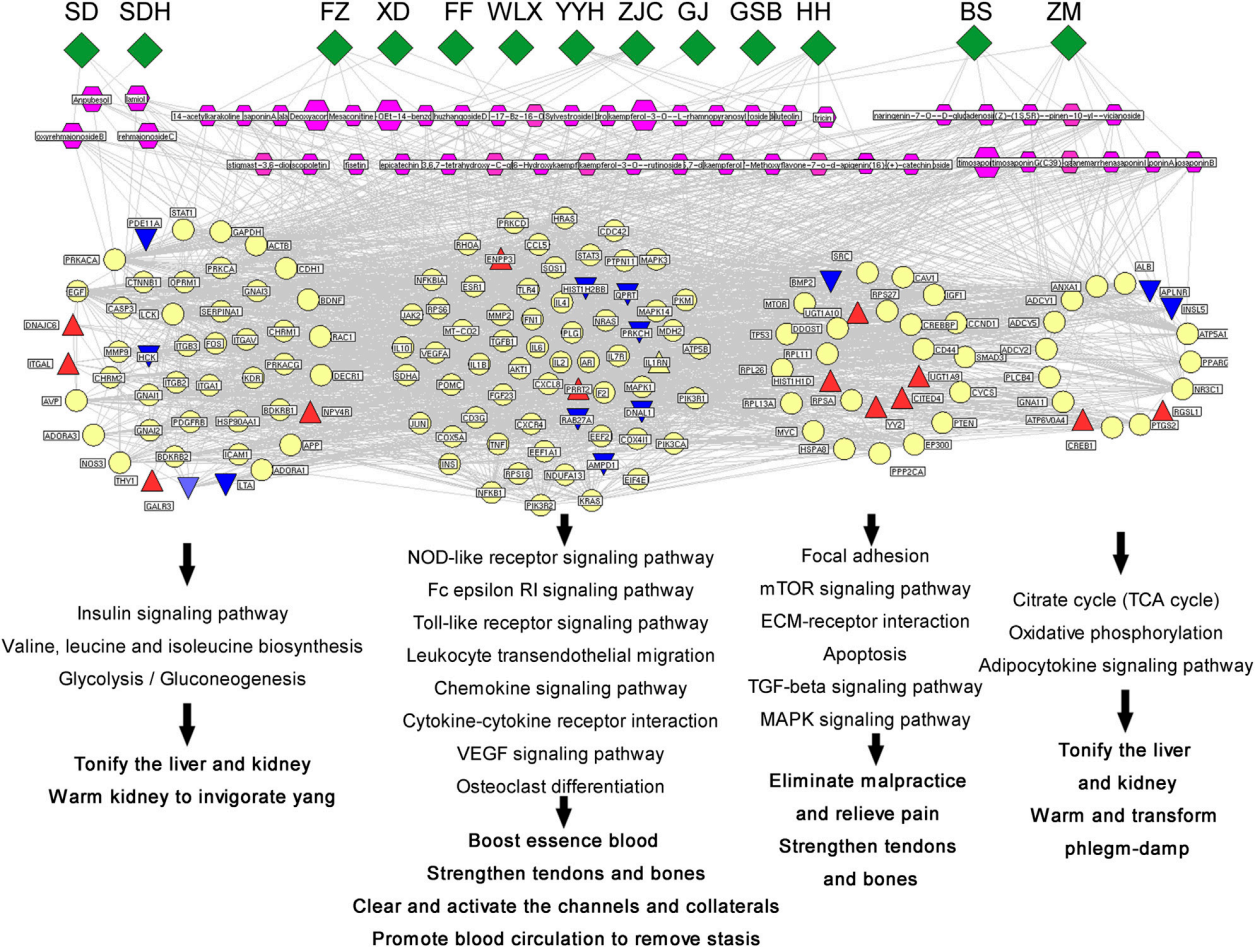
Network of “Herbal Medicines of WBT-Key Active Components-Core Modules-Traditional efficacy” was visualized using NaviGator v3.0.

The constituents that showed high-frequency binding to hub targets and had high content were selected as the key constituents (the target frequency and content were higher than the corresponding medians). A total of 48 key active constituents were selected, which corresponded to 13 herbal medicines. The detailed information is shown in Supplementary Excel S10. A multidimensional association network of “Herbal Medicines of WBT-Key Active Components-Core Targets-Functional Modules-Traditional efficacy” was visualized using NaviGator v3.0, as shown in Figure 7. Among the hub targets, 13 red triangles and 13 blue triangles represented the genes upregulated or downregulated, respectively, by WBP in the transcriptome data. These hub targets involved in different pathways were divided into four functional models corresponding to different traditional efficacies. The insulin signaling pathway; valine, leucine, and isoleucine biosynthesis; and glycolysis/gluconeogenesis were related to the tonifying of the liver and kidney as well as warming of the kidney to invigorate *yang*. The focal adhesion mTOR signaling pathway, ECM-receptor interaction apoptosis, TGF-beta signaling pathway, and MAPK signaling pathway were consistent with the elimination of malpractice, relief of pain, and strengthening of tendons and bones. The TCA cycle, oxidative phosphorylation, and adipocytokine signaling pathway were related to tonifying of the liver and kidney as well as warming and transforming of phlegm-damp. The other pathways were consistent with the boosting of blood essence, strengthening of tendons and bones, clearing and activation of the channels and collaterals, and promotion of blood circulation to remove stasis.

## Discussion

In the present study, we analyzed the chemical compositions of WBT and explore its molecular mechanisms against RA using integrative pharmacology. This is the first study to explore the active constituents and underlying mechanism of WBT in treating RA using integrative pharmacology, including rapid analysis of chemical composition, transcriptome sequencing, and network pharmacology analysis. In this study, the BPI chromatogram of WBT extracts was obtained by UPLC-QTOF-MS/MS, and 293 chemical compounds were preliminarily identified or tentatively characterized by matching the MS^E^ raw data to in-house library data using the UNIFI 1.8 software. WBT at 0.1, 1.0, and 10 μg/ml significantly reduced IL-1β and IL-6 levels in RAW264.7 cells stimulated by TNF-α. Moreover, 593 and 177 differential genes were screened between TNF-α vs. Con and between TNF-α+WBT vs. TNF-α based on transcriptome sequencing analysis. An interaction network of “Inflammatory Immune Dysregulation Genes” and “Anti-inflammatory Effect Genes” was constructed, and the hub nodes, as calculated by the topological feature values, were mainly involved in the inflammation-immune regulation module, which is closely related to the occurrence and development of RA. To explore the underlying mechanism of WBT against RA and identify its key pharmaceutically active substances, a multidimensional association network of Real-World Medicines of WBT-Key Active Components-Core Targets-Functional Modules-RA Pathology” was visualized using NaviGator v3.0. A total of 48 key active constituents were obtained based on their high-frequency binding to hub targets and contents in WBT, and 135 hub corresponding genes were selected, which may be the putative targets of WBT in treating RA. Functionally, the 135 putative targets were significantly associated with the inflammatory immune response regulation module, energy metabolism regulation module, and cell function regulation module, corresponding to their traditional efficacy.

WBT is composed of 17 herbal medicines that fit the compatibility principle of Monarch, Minister, Assistant, and Guide. We herein discuss the mechanism of WBT in treating RA according to this principle, as the 48 key active constituents were mainly involved in 13 herbal medicines: SDH and SD are Monarch drugs; YYH, XD, GJ, FZ, DH, FF, WLX, ZC, HH, and BS are Minister drugs; and ZM and BS are Assistant and Guide drugs. The other three herbal medicines and one animal drug may exert therapeutic effects through other pathways or systems than the inflammation-immune regulation system. 21 of the 48 key active constituents belong to flavonoids, 7 alkaloid, 7 steroidal saponin, 3 monoterpenoid, 3 iridoid, 2 coumarins, 1 ribonucleoside, 1 sterol, and 1 tannin. According to our experience, flavonoids may have greater potential against RA. RAW 264.7 cell which we all know is one of the media for RA testing. Inflammation is an important pathological process of RA, so we selected TNF-α as inflammatory inducer.

Interestingly, the enrichment pathways are consistent with the traditional efficacy of the corresponding herbal medicines (Figure 7.). For example, 20 hub genes corresponding to 5 chemical constituents of SDH and DH, are correlated with the regulation of glucose and lipid metabolism and blood circulation, which are consistent with the traditional effect of SDH and SD in “tonifying the liver and kidney, benefiting essence and blood”. 32 hub genes corresponding to 9 chemical constituents of FZ, FF and WLX, are mainly involved in signal transduction and inflammatory response of the nervous system, which are consistent with the traditional effect of the three herbs in “clearing and activating the channels and collaterals”. 24 hub genes corresponding to 8 chemical constituents of YYH, XD, GSB and GJ, are correlated with various regulation pathways and energy metabolism in the body, such as the citrate cycle; oxidative phosphorylation; adipocytokine signaling pathway; neurotrophin signaling pathway; insulin signaling pathway, which consistent with the “warming the kidney and strengthening *yang*” efficacy of Minister drugs. 44 hub genes corresponding to 19 chemical constituents of ZC and BS, are mainly involved in inflammatory immune regulation pathways, signal transduction, and neuroinflammatory response, which are related to the effects of warm channels and freeing of vessels, invigorating blood circulation and dispersing stasis. 48 hub genes corresponding to 14 chemical constituents of ZM and BS, were mainly involved in the regulatory pathways of nutrients and energy metabolism, blood circulation regulation pathway, which were related to the overall regulation of physical fitness and the enhancement of the effects of other drugs.

In the transcriptome sequencing experiment, we found that IL-13Rα2, Tnfaip3, Tnfrsf14, and Tnfrsf9 were upregulated in TNF-α-stimulated RAW264.7 cells, which was related with the development of inflammation (Wilson et al., 2011). The expression of Il1rn was upregulated in WBT + TNF-α-stimulated RAW264.7 cells, suggesting WBT could ameliorate inflammatory conditions stimulated by TNF-α (Matsuki et al., 2005; Hada et al., 2020). These findings were consistent with ELISA results about IL-1β and IL-6. Moreover, IL-1β and IL-6 were involved in 4 prediction pathways, including cytokine-cytokine receptor interaction, TNF signaling pathway, NOD-like receptor signaling pathway, and Toll-like receptor signaling pathway.

There were two putative genes among the hub targets of WBT, which were also the drug effect genes identified by sequencing, namely hematopoietic cell kinase (HCK) and bone morphogenetic protein 2 (BMP2). The expression level of HCK was significantly downregulated, whereas that of BMP2 was remarkably upregulated after the treatment of RAW264.8 cells with WBT. HCK, a member of the Src family of non-receptor tyrosine kinases, is primarily expressed in myeloid cells and B lymphocytes. Moreover, HCK participates in the regulation of immune function by binding to the FC terminal of immunoglobulin (Ernst et al., 2002). HCK is highly expressed in macrophages, and its expression is further augmented during macrophage activation. HCK has been reported to be involved in various inflammatory reactions (English et al., 1993). The results of transcriptome sequencing showed that after inflammation was induced by TNF-α, cellular immunity was enhanced, and the expression level of was significantly increased. After WBT intervention, however, HCK expression level was effectively inhibited. BMP2, a member of the transforming growth factor-β (TGF-β) superfamily (Koseki et al., 2002), plays an important role in the recruitment and differentiation of undifferentiated mesenchymal cells and osteoblasts (Lyons et al., 1995; Deng et al., 2008). In the early stage of bone formation, BMP2 not only induced undifferentiated stromal cells to accumulate into bone-forming centers and differentiate into osteogenic cells but also reversed the differentiation of fibroblasts, myoblasts, and bone marrow basal cells into osteoblasts. For osteoblasts, BMP2 maintains its unique cell phenotype, induces an increase in osteoblast markers, and promotes extracellular matrix calcification. In the late stage of bone formation, BMP2, as an osteoclast differentiation factor, directly or indirectly stimulates osteoclast differentiation together with other osteoclast differentiation supporting factors, and participates in bone reconstruction. It has been reported that the expression of BMP-2 decreases upon bone and cartilage destruction in the pathogenesis of RA (Sun et al., 2019). After intervention with anchoring agents or other cytokine inhibitors, the expression of BMP-2 increased significantly as the of the disease progressed, which was consistent with the transcriptome sequencing results of WBT in this study.

Although the current study reveals these important findings, there are still several potential limitations. Firstly, it is difficult to determine whether the correlation between herbs and their corresponding targets is direct or indirect. Secondly, it is difficult to confirm whether the interaction between herbs and their corresponding targets is positive or negative. Therefore, further experiments are needed to verify the results of this preliminary study.

## Conclusion

In summary, the 48 key active constituents contained in WBT may attenuate the major pathological changes in RA through their 135 candidate targets, which were involved the inflammation-immune regulation system, energy metabolism regulation module, and cell function regulation module. TCMIP v2.0 undoubtedly accelerates the process of chemical composition identification and network analysis, and transcriptome sequencing increases the accuracy of network prediction. This research strategy provides an efficient way to analyze chemical constituents and explore the pharmacological mechanism of TCM.

## Data Availability

The datasets presented in this study can be found in online repositories. The names of the repository/repositories and accession number(s) can be found below: https://www.ncbi.nlm.nih.gov/geo/query/acc.cgi?acc=GSE165272
